# Porosity Effect on Thermal Properties of Al-12 wt % Si/Graphite Composites

**DOI:** 10.3390/ma10020177

**Published:** 2017-02-14

**Authors:** José-Miguel Molina, Alejandro Rodríguez-Guerrero, Enrique Louis, Francisco Rodríguez-Reinoso, Javier Narciso

**Affiliations:** 1 Instituto Universitario de Materiales, Universidad de Alicante, Apdo 99, Alicante E-03080, Spain; jmmj@ua.es (J.-M.M.); enrique.louis@ua.es (E.L.); reinoso@ua.es (F.R.-R.); 2 Departamento de Química Inorgánica, Universidad de Alicante, Apdo 99, Alicante E-03080, Spain; alejandro.rodriguez@ua.es; 3 Departamento de Física Aplicada, Universidad de Alicante, Apdo 99, Alicante E-03080, Spain; 4 Unidad Asociada del Consejo Superior de Investigaciones Científicas, Universidad de Alicante, Apdo 99, Alicante E-03080, Spain

**Keywords:** graphite particles, porosity, gas pressure infiltration, thermal conductivity, coefficient of thermal expansion

## Abstract

The effect of porosity on the thermal conductivity and the coefficient of thermal expansion of composites obtained by infiltration of Al-12 wt % Si alloy into graphite particulate preforms has been determined. Highly irregular graphite particles were used to fabricate the preforms. The thermal conductivity of these composites gradually increases with the applied infiltration pressure given the inherent reduction in porosity. A simple application of the Hasselman-Johnson model in a two-step procedure (that accounts for the presence of both graphite particles and voids randomly dispersed in a metallic matrix) offers a good estimation of the experimental results. As concerns the coefficient of thermal expansion, the results show a slight increase with saturation being approximately in the range 14.6–15.2 × 10^−6^ K^−1^ for a saturation varying from 86% up to 100%. Results lie within the standard Hashin-Strikman bounds.

## 1. Introduction

Metal/carbon composites are a family of materials currently used in several applications such as electrical contactors [1,2], sliding contacts [3], automotive pieces [4,5,6] and plasma facing components in fusion reactors [7,8]. One of their most recent applications catching the attention of many researchers is as heat sink elements for electronics, given their high thermal conductivity and a coefficient of thermal expansion that can be easily matched to that of materials used in microelectronic systems [9,10,11].

These composites are usually fabricated by infiltration of the metal into a preform obtained by partial sintering or packing of graphite particles. Since graphite is generally poorly wetted by molten metals [12], pressure is needed to assist infiltration. In recent contributions, the authors have studied the capillary phenomena that govern the infiltration process of Al-12Si alloys in continuous graphite preforms [13] and packed performs of graphite particles [6,14]. These studies confirm, by means of drainage curves (plots of degree of metal filling, or saturation, versus applied pressure), that infiltration takes place progressively with applied pressure and time at pressure. As a consequence, porosity in a carbon/metal composite processed at a fixed pressure appears as an unavoidable phase inherently linked to poor wetting at the interface. In spite of the high interest in carbon/metal composite materials, little attention has been paid to the effects of the applied pressure (hence porosity) on the properties of final materials.

Amongst the two thermal properties currently evaluated in materials with interest in electronics, thermal expansion and thermal conductivity, the former does present little or null dependence on the presence of pores, as has been proved for different systems [15,16,17]. Porosity, on the contrary, may strongly decrease the thermal conductivity of a material and the study of its influence becomes an important issue to be addressed.

In the present study, Al-12 wt % Si/graphite composites have been obtained by infiltration of the metal into performs of highly irregular graphite particles of three different sizes. The infiltration pressure has been varied in order to obtain composites with various degrees of porosity. Both the thermal conductivity (TC) and the coefficient of thermal expansion (CTE) of the composites have been measured. A simple two-step application of the Hasselman-Johnson model rationalizes the effect of the porosity on the measured thermal conductivity values. On the other hand, the Hashin-Strikman bounds work nicely to determine the range over which the CTE is expected to vary.

## 2. Materials and Methods

### 2.1. Materials and Fabrication Procedures

**Figure 1:**
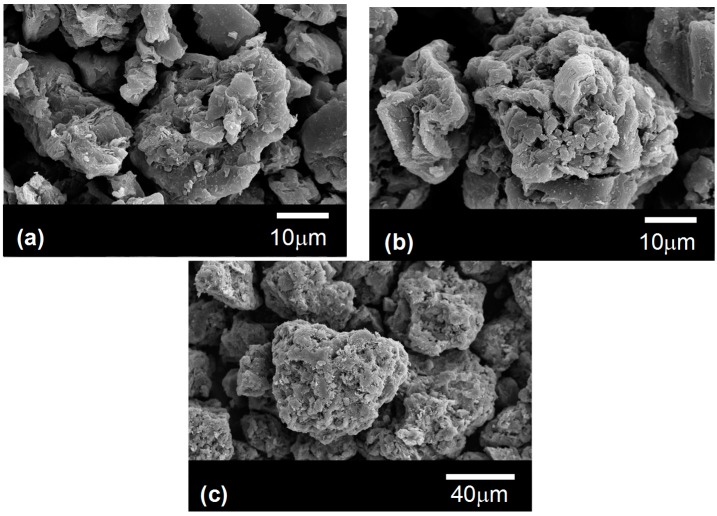
SEM images of the three types of graphite particles used in this work named G1 (**a**); G2 (**b**) and G3 (**c**).

Graphite particles of three different sizes were used in this study. They were kindly supplied by Schunk Kohlenstofftechnik GmbH (Heuchelheim, Germany). The three particles are fabricated by the same method of milling graphite blocks obtained by the subsequent steps of cold isostatic pressurization, carbonization and graphitization at 2500 °C. The degree of graphitization is around 78%. More information on the characteristics of these particles can be found in [6]. SEM images of the graphite particles can be observed in Figure 1 and their main characteristics are gathered in Table 1. The high specific surface areas of these particles, a factor of 5 higher than that of typical ceramic particles [18], are a consequence of their highly irregular shapes. The eutectic Al-Si alloy, which contains 12 wt % of silicon Al-12 wt % Si (hereafter referred as Al-12Si), used for the infiltration experiments was kindly supplied by Leichtmetall Kompetenzzentrum Ranshofen GmbH (Ranshofen, Austria).

The particles were packed in quartz crucibles of 17 mm inner diameter by means of alternative strokes of a piston and vibrations. A piece of solid Al-12Si alloy was placed inside the tube and on top of the graphite packed preform. Infiltration was carried out in a chamber described elsewhere [19,20,21]. Basically, it consists of a chamber that allows pressurization up to 5 MPa and directional solidification. Before heating, vacuum was applied until a pressure of 0.1 mbar was reached. Then the chamber was heated up to 670 °C and pressurized at different pressures over threshold (see [6] for details of threshold pressure for infiltration of graphite particles with Al-12Si alloy). The infiltration time was fixed at two minutes and finally the system was cooled directionally under pressure (see [21] for more details). The extent of metal filling was determined on each infiltrated sample by means of densitometry, once the particle volume fraction was determined as explained in [18].

### 2.2. Measurement of Thermal and Electrical Conductivity and Coefficient of Thermal Expansion

The thermal conductivity of the composites was measured by means of a relative steady-state technique, in an experimental set up assembled in our laboratories (see [20] for a detailed explanation). The overall uncertainty of the measured thermal conductivities is calculated to be about ±5%. The electrical conductivity of the pore-free metal remaining non infiltrated on top of the samples was measured by means of an Eddy-current apparatus (SIGMATEST 2.069) purchased from FOERSTER INSTRUMENTS INC., Pittsburgh, PA, USA). Its accuracy is about 0.1 × 10^−6^ Ω^−1^·m^−1^.

A thermomechanical analyser (TMA 2940, TA Instruments, Trevose, PA, USA) was used to obtain the thermal response curves from which the coefficient of thermal expansion was derived. Samples of approximately 5 mm in length were cut from the infiltrated composites using a low speed saw, and they were all subsequently polished. Measurements were carried out at an applied force of 0.05 N, under nitrogen atmosphere, and in the temperature range 298–573 K (heating and cooling rates were 3.00 K/min). The samples were subjected to at least four heating and cooling cycles to remove large residual stresses, if any, developed during processing of the composite. It is worth noting that the hysteresis in the thermal cycle is highly sensitive to the experimental conditions, particularly to the heating and cooling rates, sample size and shape, and the period of time elapsed between the point at which the maximum temperature was reached and the initiation of cooling. The latter was kept close to zero in the present experiments.

## 3. Results

The volume fraction attained in the graphite compacts is approximately 0.53 for the three particles, which is a reasonable value for a preform formed by random packing of non-spherical particles (see [18] for a comprehensive report of experimental data obtained by different groups). The infiltration pressure and the attained saturation at each pressure are also shown in Table 2. 

**Figure 2:**
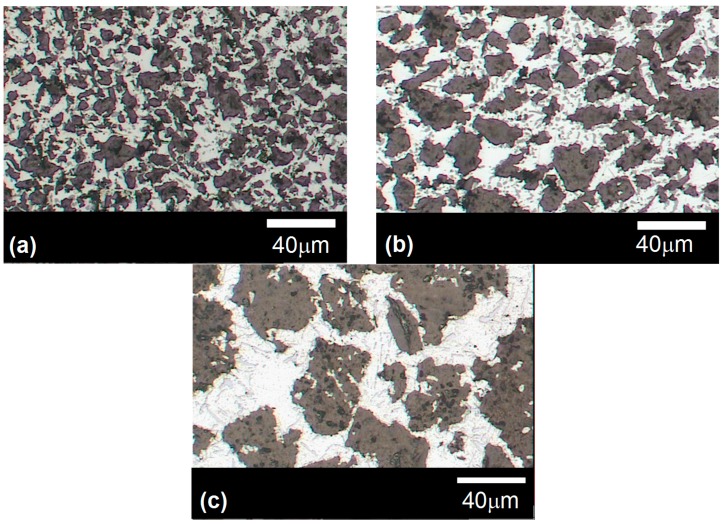
Optical microscopy images of the composites obtained by infiltration with Al-12Si of G1 (**a**); G2 (**b**) and G3 (**c**) particles corresponding to samples G1-4, G2-6 and AG-7 respectively.

As the results reported in Table 2 unambiguously show, saturation increases with applied pressure, confirming the non-wetting nature of the Al-12Si/graphite system. Figure 2 shows optical micrographs of the composites fabricated by infiltration of the graphite particles for pressures at which saturation reaches its maximum value (close to 100%).

The composites appear to have a rather homogeneous distribution of graphite particles. Moreover, there are no clear evidences of particle breaking, which makes these composites suitable for confrontation with predictive models. Focusing the attention on the values of thermal conductivity, it is apparent that this property is strongly affected by saturation, and hence porosity (Table 1). The pores present in the composite may be understood as a third phase (with a given volume fraction) consisting of a thermally isolating material. Experimental results for the CTE are also shown in Table 2. The results corroborate the little or null dependence on the presence of pores already observed in different systems [15,16,17]. This is better illustrated in Table 2 whereby a change in porosity from 14% to 0% only produces an increase in CTE from approximately 14.3 up to 15.5 × 10^−6^ K^−1^.

## 4. Discussion

### 4.1. Thermal Conductivity

Thermal conductivity in composite materials is mainly governed by the conductivity of the individual phases, their volume fraction and shape, and also by the size of the inclusion phase due to a finite metal/ceramic interface thermal resistance. Modeling of thermal conductivity of composite materials with thermally conductive inclusions has been extensively studied (see, for example, [22,23]). One of the easiest analytical models that assume a non-idealized interface between matrix and reinforcement is that proposed by Hasselman-Johnson [24]. In particular, they investigated the case of spherical particles in a pore-free infinite matrix:

(1) $$\kappa_{c}=\;\;\frac{\kappa_{m}\left[2\kappa_{m}+\kappa_{p}^{eff}+2\left(\kappa_{p}^{eff}-\kappa_{m}\right)V_{p}\right]}{2\kappa_{m}+\kappa_{p}^{eff}-\left(\kappa_{p}^{eff}-\kappa_{m}\right)V_{p}}$$

where *κ_m_* and *κ_c_* are the thermal conductivities of metal and composite and $\kappa_{p}^{eff}$ is the effective thermal conductivity of particles. *V_p_* is the volume fraction of particles. This model has been proved to give accurate predictions when particles and matrix exhibit a low ratio of conductivities [25], which is the case of graphite particles and Al-Si eutectic. The effective thermal conductivity of particles $\kappa_{p}^{eff}$ is given by:

(2) $$\kappa_{p}^{eff}=\frac{\kappa_{p}^{in}}{1+\frac{\kappa_{p}^{in}}{ah_{c}}}$$

where $\kappa_{p}^{in}$ stands for the intrinsic thermal conductivity of particles while *a* and *h_c_* are the average radius of particulate and the interfacial thermal conductance respectively.

The residual pores in composite materials processed by infiltration can be treated as non-thermally conducting inclusions in the metal. Hence, *κ_m_* in Equation (1) can be calculated with the Hasselman-Johnson model as well, conveniently adapted for dispersions of zero conductivity:

(3) $$\kappa_{m}=2\kappa_{m}^{in}\frac{1-V_{po}}{2+V_{po}}$$

where $\kappa_{m}^{in}$ is the intrinsic thermal conductivity of the metal (free of pores) and *V_po_* the volume fraction of pores in the metal. It is worth noting that Equation (3) corresponds to the well-known expression of Maxwell’s model that is applied for composites with spherical non-conducting inclusions in a conductive matrix [22].

In modeling thermal conductivity of porous composites, Equations (1) and (3) may be used consecutively for calculating the thermal conductivity of the matrix (considered as formed by Al-12Si alloy and pores) and the thermal conductivity of the overall composite (with a matrix of Al-12Si + pores and graphite inclusions as reinforcement). Applying this procedure requires a precise value of the thermal conductivity of the metal (Al-12Si). This parameter may vary considerably depending on the specific infiltration conditions of samples. However, it can be indirectly estimated by measuring the electrical conductivity of the pore-free metal remaining non infiltrated on top of the samples. Table 2 collects these measurements for all samples. The electrical conductivity increases somehow with the applied pressure. Since electrical conductivity of alloys varies with the solubility state of the alloying elements, the evolution observed in Table 2 suggests that the solidification conditions under which samples have been processed have indeed varied from sample to sample. This is not surprising given that the pressure applied in the infiltration chamber influences the cooling conditions of the equipment. In metals and alloys, the following relation between the electrical conductivity and the thermal conductivity holds,

(4) $$\kappa_{m}^{in}=L\sigma T$$

where $\kappa_{m}^{in}$ is the intrinsic thermal conductivity of the metal, *σ* the electrical conductivity, *T* the temperature and *L* the Lorentz number (approximately 2.16 × 10^−8^ WΩ/K^2^ for Al at T = 300 K [26]). In using Equation (1), two unknown parameters are needed: the intrinsic thermal conductivity of the particles $\kappa_{p}^{in}$ and the interface thermal conductance *h_c_*. Albeit graphite is a well-known and extensively characterized material, it is not easy to ascribe an intrinsic value of thermal conductivity to the graphite particles. Their purity, as well as their particular fabrication route, are important factors that considerably affect this property. According to Equation (2), the effective thermal conductivity of the particles, $\kappa_{p}^{in}$, can be rewritten as follows:

(5) $$\frac{1}{\kappa_{p}^{eff}}=\;\frac{1}{\kappa_{p}^{in}}+\;\frac{1}{ah_{c}}$$

**Figure 3:**
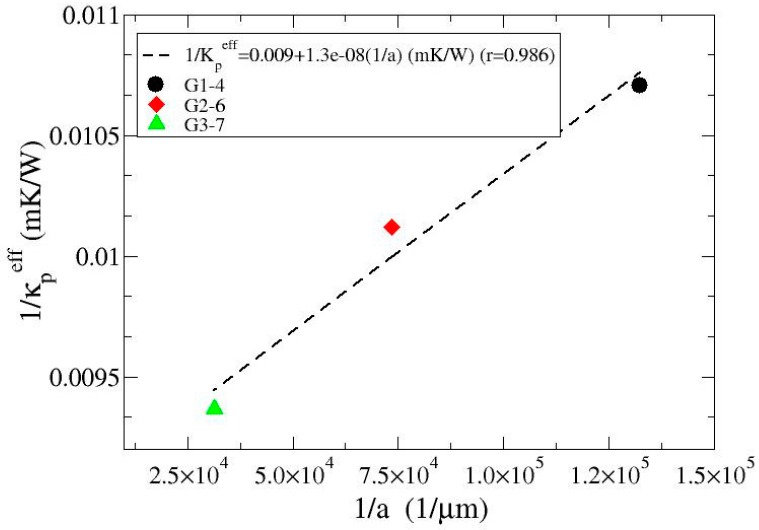
1/$\kappa_{p}^{eff}$ (inverse of the effective particle thermal conductivity) versus 1/a (inverse of the particle radius) for samples G1-4, G2-6 and G3-7 for which zero porosity is ascribed. The straight line fitted to the experimental data has a regression coefficient r of 0.986.

A plot of the inverse of the experimental effective particle conductivity versus the inverse of the particle radius is shown in Figure 3. The data can be satisfactorily fitted by means of a straight from which the intrinsic thermal conductivity of the particles $\kappa_{p}^{in}$ and the interfacial conductance *h_c_* is derived (see Equation (5)). In order to avoid effects associated to porosity, Figure 3 gathers only data for samples infiltrated at maximum pressure (G1-4, G2-6, G3-7). The values derived from the linear fitting are $\kappa_{p}^{in}$ = 110 W/mK and *h_c_* = 7.7 × 107 W/m^2^·K. Introducing these values in Equations (1) and (3), the thermal conductivity of the composites with various degrees of porosity can be calculated.

**Figure 4:**
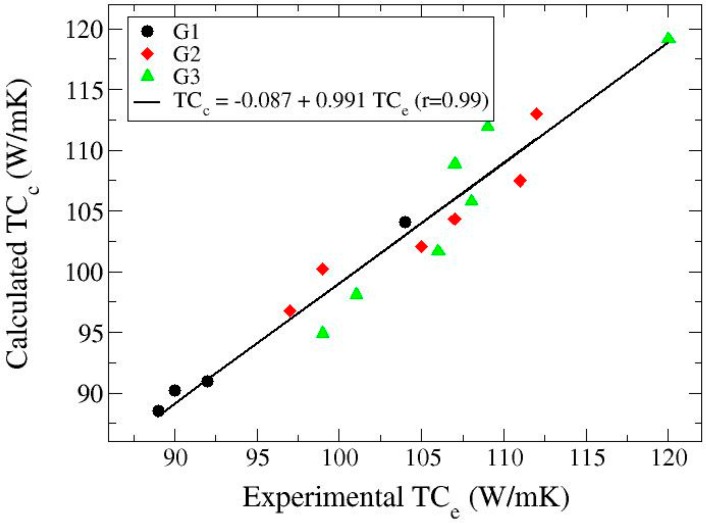
Plot of the thermal conductivity calculated with the two-step Hasselman-Johnson model versus that determined experimentally for all samples in Table 2. Symbols: black circles (G1 particles), red diamonds (G2 particles) and green triangles (G3 particles). The line is a linear fitting to all data (r is the regression coefficient).

Figure 4 shows the confrontation of the calculated thermal conductivity versus the experimental data for the different samples fabricated. In accordance with expectations, the predictions have good correspondence with the measured values. This indicates that the two-step procedure of the Hasselman-Johnson model followed in this work is robust enough to account for the porosity in composite samples as well as giving access to both the intrinsic value of particle thermal conductivity and the interfacial conductance characteristic of the material under given processing conditions. Actually, the values derived for those parameters are in good accordance with literature. On one hand, the value of 110 W/mK represents the intrinsic thermal conductivity of the graphite particles with a given internal porosity (indicated in Table 2) that depends on the particle type. If we subtract the effect of internal pores by applying a convenient modified form of Equation (3), we obtain values of 105–120 W/mK for the pore-free graphite material. These values are coherent with the specifications given by the producer deduced from measurements of thermal conductivity of bulk materials obtained by particle compression. On the other hand, the value of the interfacial conductance *h_c_* may be estimated with various models, one of the simplest being the acoustic mismatch model [23]. This model treats the interface heat transfer in terms of continuum mechanics by calculating the probability of an incident phonon to pass the interface. *h_c_* is calculated to be:

(6) $$h_{c}≅\;\frac{1}{2}C_{p}\left(\rho_{m}c_{m}\right){\left(\frac{c_{m}}{c_{p}}\right)}^{2}\frac{\rho_{m}c_{m}\rho_{p}c_{p}}{{\left(\rho_{m}c_{m}+\;\rho_{p}c_{p}\right)}^{2}}$$

where *C_p_* is the specific heat of metal, *ρ_m_* and *ρ_p_* are the densities and *c_m_* and *c_p_* the phonon velocities of metal and ceramic particles, respectively. Taking *ρ_m_* = 2700 kg/m^3^, *C_p_* = 895 J/kg·K and *c_m_* = 3595 m/s for the metal [26] (considered here as pure aluminum, given the lack of data for Al-Si alloys) and *c_p_* = 1500 m/s for graphite [27], we obtain *h*_c_ = 1.4 × 10^8^ W/m^2^·K, which is very close to the value obtained experimentally.

### 4.2. Coefficient of Thermal Expansion

**Figure 5:**
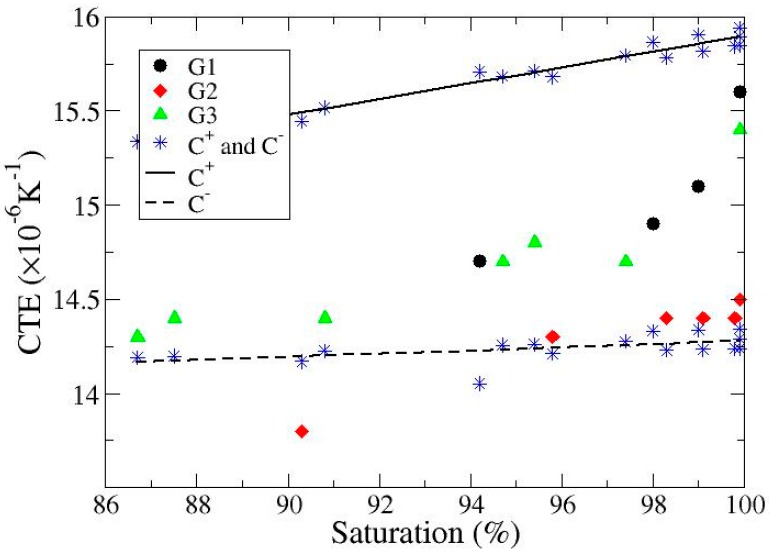
Experimental data for the coefficient of thermal expansion of the composites investigated in this work (G1, G2 and G3) versus saturation (degree of filling). Upper and lower bounds (see text) are also shown.

All sophisticated treatments of the CTE of composites are based in thermo-elasticity theory. Schapery’s model [28,29,30] gives upper (+) and lower (−) bounds to the CTE. The specific expression for the former is,

(7) $$\alpha_{c}^{\left(+\right)}=\alpha_{p}+\left(\alpha_{m}-\alpha_{p}\right)\frac{K_{m}\left(K_{p}-K_{c}^{\left(-\right)}\right)}{K_{c}^{\left(-\right)}\left(K_{p}-K_{m}\right)}$$

where $K_{c}^{\left(-\right)}$ is the lower bound of the volumetric modulus of the composite and *K_p_* and *K_m_* the volumetric moduli of the reinforcement and the matrix, respectively. Bounds to the volumetric moduli of the composite are calculated with the Hashin-Strikman model. In particular, the lower bound $K_{c}^{\left(-\right)}$ is given by,

(8) $$K_{c}^{\left(-\right)}=K_{m}+\frac{V_{m}}{\frac{1}{K_{p}\;-\;K_{m}}\;+\;\frac{V_{m}}{K_{m}\;+\;\frac{4}{3}\mu_{m}}}$$

where *μ_m_* is the shear modulus of the metal. The upper bound to the bulk modulus is obtained by interchanging the subscripts *m* and *p* everywhere in Equation (8) and, when inserted in Equation (7), this gives the lower bound on the CTE. In calculating bounds for the present case, we included the effects of porosity on the elastic (or Young) modulus of the metal matrix as in [31],

(9) $$E=\;E_{m}{\left(1-V_{po}\right)}^{23/12}$$

in addition, we took 0.32 for the Poisson coefficient of polycrystalline graphite [32]. Results are shown in Figure 5. It is noted that with a single exception, experimental data are within the calculated bounds.

## 5. Conclusions

The conclusions derived from the experimental work and the theoretical analysis presented in this paper are: (i) thermal conductivity of Al-12Si/graphite materials processed by fixed-pressure infiltration is considerably affected by the presence of residual pores; (ii) the thermal conductivity of these composites can be predicted by using the Hasselman-Johnson model in a two-step procedure in which pores are treated as inclusions of zero thermal conductivity; (iii) as already found in a variety of systems, porosity scarcely affects the coefficient of thermal expansion; and finally; (iv) with the exception of a single case (out of 17), experimental data for the coefficient of thermal expansion lie within Hashin and Strikman bounds.

## Figures and Tables

**Table 1:** Main characteristics of the graphite particles: average diameter (D, taken equal to D(4,3), see [18]), span of the size distribution, density ρ and percentage of internal porosity (IP, in %). The span is defined as (D(90)-D(10))/D(50), where D(x) is the diameter below which x % of the particulates are found. The percentage of Internal Porosity (IP) and the Specific surface areas (S) are also given.

Particle	D (μm)	Span	ρ (g/cm^3^)	IP (%)	S (m^2^/kg)
G1	15.1	1.39	2.24	0.20	7720
G2	27.2	0.99	2.20	1.98	3620
G3	64.0	0.94	2.18	2.87	950

**Table 2:** Thermal conductivity (TC) (in W/mK) of the composites obtained with graphite particles and Al-12Si alloy for different infiltration pressures (P) (in kPa). The TC (W/mK) calculated with the two-step Hasselman-Johnson model is given in parenthesis. S_a_ is the saturation (percentage of metal filling; porosity in % is given by 100-S_a_). EC is the electrical conductivity (in MS/m) of the remaining non-infiltrated metal on top of the preform and CTE stands for the coefficient of thermal expansion (×10^−6^ K^−1^).

Sample	P_i_	S_a_	TC	EC	CTE
G1-1	2360	94.2	89 (86)	14.0	14.7
G1-2	3130	98	90 (90)	14.0	14.9
G1-3	3700	99	92 (91)	14.1	15.1
G1-4	4200	99.9	104 (104)	18.0	15.6
G2-1	1070	90.3	97 (97)	16.6	13.8
G2-2	1390	95.8	99 (100)	16.4	14.3
G2-3	2100	98.3	105 (102)	16.4	14.4
G2-4	2650	99.1	107 (104)	17.0	14.4
G2-5	3320	99.8	111 (108)	17.9	14.4
G2-6	4200	99.9	112 (113)	19.8	14.5
G3-1	730	86.7	99 (95)	15.8	14.3
G3-2	980	87.5	101 (98)	16.8	14.4
G3-3	1580	90.8	106 (102)	17.2	14.4
G3-4	2190	94.7	108 (106)	17.6	14.7
G3-5	2800	95.4	107 (109)	18.5	14.8
G3-6	3300	97.4	109 (112)	19.0	14.7
G3-7	4200	99.9	120 (119)	21.0	15.4
